# The prognostic impact of BMI on colorectal cancer is stratified by tumor location

**DOI:** 10.3389/fonc.2022.987518

**Published:** 2022-11-07

**Authors:** Zixi Zhang, Xueli Yan, Yan Lu, Xiaowen Guo, Min Jiao, Weizhong Wang, Boqian Sun, Yi Zhou, Qinglin Hu, Dake Chu

**Affiliations:** ^1^ Department of Gastroenterology, The First Affiliated Hospital of Xi’an Jiaotong University, Xi’an, China; ^2^ Department of Oncology, The First Affiliated Hospital of Xi’an Jiaotong University, Xi’an, China; ^3^ State Key Laboratory of Cancer Biology, Department of Gastrointestinal Surgery, Xijing Hospital of Digestive Diseases, Air Force Medical University, Xi’an, China; ^4^ Department of Hospital Management, Affiliated Hospital of Beihua University, Jilin, China; ^5^ Department of Gastrointestinal Surgery, Tianjin People’s Hospital, Tianjin, China; ^6^ Department of General Surgery, The First Affiliated Hospital of Chengdu Medical College, Chengdu, China

**Keywords:** colorectal cancer, body mass index (BMI), tumor location, overall survival, disease specific survival (DSS)

## Abstract

**Purpose:**

Recent studies have revealed the contrasting prognostic roles of body mass index (BMI) and tumor location in colorectal cancer (CRC). Given that right- and left-sided CRC may exhibit inverse effects on outcome and body weight, the present study aimed to examine whether the prognostic value of BMI and tumor location could be reciprocally stratified.

**Methods:**

This prospective, observational study recruited 4,086 patients diagnosed with stage III CRC from five independent clinical centers in China. The association of patients’ outcomes with BMI and tumor location was evaluated hierarchically by Kaplan–Meier and Cox proportional-hazards models.

**Results:**

Although BMI was not associated with overall outcome, the association was significantly modified by tumor location. Among left-sided tumors, obesity and overweight were significantly associated with adverse overall survival (OS) and disease-specific survival (DSS). In contrast, among right-sided tumors, overweight was significantly associated with more favorable OS and DSS compared with the normal-weight group. The association of survival with tumor location did not reach statistical significance. However, hierarchical analysis by BMI revealed that left-sided tumors were associated with more favorable outcomes in the normal-weight group, while there was no statistically significant difference in the overweight or obese group.

**Conclusions:**

BMI and tumor location may have opposing effects on CRC prognosis, when stratified by each other, after adjusting for other known prognostic factors. These findings are the first to show the interactive prognostic impact of BMI and tumor location, which could be relevant to the stratification of patient management.

## Introduction

Colorectal cancer (CRC) is one of the most common malignancies worldwide (1–3). Previously, it occurred primarily in western countries whose populations frequently exhibit CRC risk factors, including obesity (2, 4–9). However, incidence rates of CRC have recently stabilized or decreased in most developed countries (3, 10–13). In contrast, the incidence rates of CRC are rapidly increasing in eastern Asia, including China, Japan, and South Korea (3, 14–16). In China, there has been a two- to four-fold increase in the incidence of CRC since the 1980s (17–19), which can be explained, at least in part, by changes in lifestyle factors (16, 19, 20).

Previous studies have found an association between high body mass index (BMI) and an elevated risk of developing CRC (11, 21–30). However, data on the potential effect of BMI on the prognosis of CRC are scarce, and the limited publications available on this topic have shown conflicting results (4, 31–37). In the National Cancer Institute (NCI)-sponsored Cancer and Leukemia Group B (CALGB) study, neither BMI nor weight change was found to be associated with outcome in patients with stage III colon cancer (31). In the National Surgical Adjuvant Breast and Bowel Project (NSABP), only severe obesity was found to be associated with colon cancer outcomes (32). However, another NCI-sponsored investigation found obesity to be an independent prognostic variable in colon cancer survivors (36). Two studies that focused on female patients obtained differing results regarding the prognostic value of BMI in CRC (33, 38).

Recently, the association between tumor location and the prognosis of human colorectal cancer has been investigated, and the studies to date have produced conflicting results. One study involving the Surveillance, Epidemiology, and End Results Program (SEER) database showed a significant increase in mortality for right-sided tumors compared with left-sided ones (39, 40). In contrast, another study using the SEER database found no overall difference between right- and left-sided colon cancers when stratified by tumor stage (41), and an investigation based on the NCIC CO.17 trial demonstrated that tumor location is not a prognostic factor in CRC (42). More recently, a study on metastatic CRC found that patients with left-sided tumors had superior overall and progression-free survival compared with right-sided ones (43). Considering that right- and left-sided tumors may exhibit different effects on body weight and even on outcomes, we hypothesized that there might be interactions between BMI and tumor location in the determination of prognosis (44, 45). Therefore, the intrinsic prognostic role of BMI and tumor location in CRC may be better understood after stratified analysis.

In the present study, we used stratified analysis to investigate the associations between BMI, tumor location, and outcomes in a large cohort of patients with resected stage III CRC.

## Patients and methods

### Study cohort

The study protocols of the present investigation were approved by the review boards of the participating institutions and conducted in accordance with the Declaration of Helsinki and Good Clinical Practice Guidelines. This prospective cohort study was based on the Nutrition and Lifestyle Study Cohort of Colorectal Cancer in China (NCT02215642). The study population consisted of a series of participants consecutively diagnosed with stage III colorectal cancer in four separate clinical centers—namely, the Fourth Military Medical University, Tianjin Union Medical Center, Beihua University, and Chengdu Medical University—between January 2004 and December 2008. All included participants had pathologically confirmed CRC and received surgical resection with curative intent, as well as effective 5-fluorouracil-based adjuvant chemotherapy. Participants with any history of cancer (other than skin cancer), non-adenocarcinoma, inflammatory bowel disease, as well as those who received either ineffective chemotherapy or no treatment, had inaccurate follow-up, had missing BMI data, or who withdrew from the survey were excluded. The final parent study cohort comprised 4,086 patients who met all of the inclusion criteria. An independent data review was performed for quality assurance, and all of the entered data were checked for accuracy by administrative research staff.

### Assessment of BMI and tumor location

To ensure the consistency of BMI values, weight (in kilograms) and height (in meters) were measured and recorded by trained staff at the point of preoperative assessment for each patient. BMI was categorized according to the World Health Organization (WHO) classification for Asian populations as normal weight (18.5 kg/m^2^ ≤ BMI < 23.0 kg/m^2^), overweight (23.0 kg/m^2^ ≤ BMI < 27.5 kg/m^2^), or obese (BMI ≥ 27.5 kg/m^2^). Participants with a BMI of <18.5 kg/m^2^ were defined as underweight and excluded. Self-reported weight was also recorded at the same time of BMI measurement, and it was found to have a strong correlation of 0.92 (*p* < 0.001) with that measured by clinical staff. Therefore, weight loss was calculated by subtracting the patient’s self-reported 12-month presurgical weight from the weight measured for BMI.

Tumor location was identified from medical records. Tumors proximal to the splenic flexure, such as those occurring in the cecum, ascending colon, or transverse colon, were classified as right-sided tumors, while those distal to the splenic flexure, such as tumors occurring in the descending colon, sigmoid colon, or rectum, were classified as left-sided tumors (43). Baseline performance status was recorded according to the Eastern Cooperative Oncology Group Performance Status Scale.

### Follow-up and clinical endpoints assessment

All participants were followed up every 3 months by telephone conversation with the participant or their first-degree relatives. For participants who died during follow-up, notice of the death was ascertained by reports from the family, and the details were verified by reviewing death certificates. The cause of death, which was assigned by physicians blinded to information regarding lifestyle exposure, was obtained from a formal medical file or an official death certificate. For the primary purpose, we included overall survival (OS) and disease-specific survival (DSS) as the clinical endpoints. OS was defined as the time that elapsed from surgery to the date of death from any cause. DSS was defined as the time that from surgery to death related to CRC, censored at the date of death resulting from postoperative complications or other nonmalignant causes.

### Statistical analysis

Statistical analysis was conducted using SPSS statistical software (version 13.0). χ^2^ and Wilcoxon rank-sum tests were used to test for an association between the categorical variables of the different groups. The survival curves were determined using the Kaplan–Meier method, and differences in the survival distributions were evaluated using the log-rank test. The factors potentially related to survival were analyzed *via* Cox’s proportional hazards modeling to determine which of them might have had a significant influence on survival. To determine whether the tumor location and BMI modified outcomes, the categorical BMI, tumor location, and data on weight loss were included in the Cox model. Differences with a *p*-value of 0.05 or less were considered to be significant, and all of the *p*-values were determined using two-sided tests.

## Results

### Study cohort characteristics

**Figure 1:**
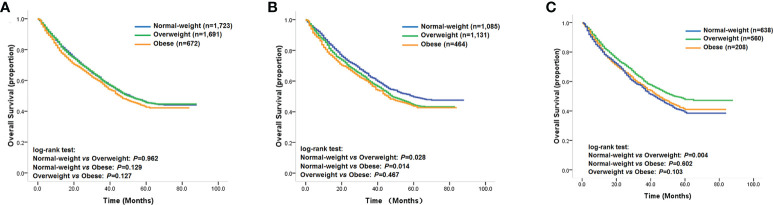
**(A)** Kaplan–Meier survival curves of the overall study cohort; **(B)** Kaplan–Meier survival curves of patients with left-sided tumors; **(C)** Kaplan–Meier survival curves of patients with right-sided tumors.

The baseline clinicopathological data for the study cohort is shown in **Table 1**. According to our predetermined BMI categories, 672 patients (16%) were obese, 1,691 patients (41%) were overweight, and 1,723 patients (42%) were of normal weight. Analysis of the baseline characteristics showed that a higher percentage of patients with right-sided tumors were female compared with those with left-sided tumors (42% vs. 38%; *p* = 0.004). Patients with right-sided tumors were significantly older than those with left-sided tumors (39% vs. 36% > 60 years old; *p* = 0.042). Patients with right-sided tumors were also less likely to be obese or overweight than those with left-sided tumors (15% vs. 17% and 40% vs. 42%, respectively; *p* = 0.007). In addition, right-sided tumors were also less likely to be well-differentiated than left-sided tumors (11% vs. 14%; *p* = 0.004). Although no significant prognostic difference was found among the obese, overweight, and normal weight groups (**Figure 1A**), statistical analysis revealed that the multiplicative interaction of BMI and tumor location was significant for OS (*p* = 0.001), indicating that the prognostic value of BMI differed according to tumor location. We therefore further investigated the association between BMI and OS/DSS within left-sided and right-sided tumors.

**Table 1:** Clinical characteristics of patients according to smoking status.

		Smoking status				
Variable	*n*	Never		Ever		*p*
		No.	%	No.	%	
Total	4,136	2,664	64	1,472	36	
**Sex**						< 0.001^*^
Male	2,512	1,172	47	1,340	53	
Female	1,624	1,492	92	132	8	
**Age at diagnosis**						0.719^*^
≤ 60	2,572	1,662	65	910	35	
> 60	1,564	1,002	64	562	36	
**BMI**						0.070^*^
Normal weight	1,750	1,120	64	630	36	
Overweight	1,700	1,124	66	576	34	
Obese	686	420	61	266	39	
**Performance status**						0.197^*^
0	3,144	2,042	65	1,102	35	
1–2	992	622	63	370	37	
**Weight loss**						0.193^*^
≤ 10%	3,332	2,162	65	1,170	35	
> 10%	804	502	62	302	38	
**Tumor location**						0.134^*^
Right	940	614	65	326	35	
Left	1,286	850	66	436	34	
Rectum	1,910	1,200	63	710	37	
**Tumor size**						0.217^*^
≤ 3.0 cm	900	564	63	336	37	
> 3.0 cm	3,236	2,100	65	1,136	35	
**Differentiation status**						0.449^*^
Well	984	632	64	352	36	
Moderately	1,792	1,172	65	620	35	
Poorly	1,360	860	63	500	37	
**Depth of invasion**						0.180^*^
T_1_ + T_2_	1,432	942	66	490	34	
T_3_ + T_4_	2,704	1,722	64	982	36	
**Lymph node metastasis**						0.503^*^
Absent (N0)	1,792	1,144	64	648	36	
Present (N1–3)	2,344	1,520	65	824	35	
**Distant metastasis**						0.928^*^
Absent (M0)	3,684	2,372	64	1,312	36	
Present (M1)	452	292	65	160	35	
**TNM stage**						0.204^*^
I	820	498	61	322	39	
II	972	626	64	346	36	
III	1,892	1,228	65	664	35	
IV	452	292	65	160	35	
**Serum CEA**						0.862^*^
≥ 5 ng/ml	1,656	1,064	64	592	36	
< 5 ng/ml	2,480	1,600	65	880	35	

^*^p-value when expression levels were compared using Pearson test.

CEA, carcinoembryonic antigen.

### Prognostic value of BMI in left-sided tumors

Among patients with left-sided tumors, Kaplan–Meier analysis showed that obese and overweight patients had worse OS compared with normal-weight patients, while no significant prognostic differentiation was found between obese and overweight patients (**Figure 1B**). The unadjusted hazard ratios (HR) for the overweight and obese patients with left-sided tumors were 1.14 (95% CI, 1.01–1.28; *p*=0.029) and 1.20 (95% CI, 1.04–1.40; *p*=0.016), respectively, compared to normal-weight patients. Next, we evaluated the effect of BMI on DSS status. The univariate analysis showed a similar trend as the OS analysis in that categorical BMI was significantly associated with DSS; i.e., patients with normal weight had favorable DSS compared with those who were overweight or obese (**Table 2**). The unadjusted HRs of DSS for overweight and obese patients relative to normal-weight patients were 1.12 (95% CI, 1.00–1.25; *p*=0.061) and 1.32 (95% CI, 1.14–1.52; *p*<0.001), respectively. Among other clinicopathological characteristics, performance status, differentiation status, depth of tumor invasion, node metastasis, and distant metastasis were found to be associated outcomes of left-sided tumors (**Table 2**, *p*<0.05).

**Table 2:** Associations between BMI and clinical factors with outcome in left-sided tumors.

	Overall survival				Disease-specific survival			
	Unadjusted HR (CI 95%)	*p*	Adjusted HR (CI 95%)	*p*	Unadjusted HR (CI 95%)	*p*	adjusted HR (CI 95%)	*p*
**BMI**								
Normal weight	Ref		Ref		Ref		Ref	
Overweight	1.14 (1.01–1.28)	0.029	1.12 (0.99–1.28)	0.076	1.12 (1.00–1.25)	0.061	1.08 (0.92–1.26)	0.374
Obese	1.20 (1.04–1.40)	0.016	1.18 (1.03–1.39)	0.045	1.32 (1.14–1.52)	< 0.001	1.17 (1.03–1.33)	0.015
**Weight loss**								
≤ 10%	Ref		Ref		Ref		Ref	
> 10%	1.26 (0.93–1.71)	0.141	1.22 (0.89–1.65)	0.273	1.21 (0.90–1.64)	0.205	1.17 (0.91–1.50)	0.218
**Sex**								
Male	Ref		Ref		Ref		Ref	
Female	0.95 (0.86–1.06)	0.390	0.97 (0.87–1.08)	0.544	0.89 (0.79–1.01)	0.072	0.90 (0.79–1.01)	0.079
**Age at diagnosis**								
≤ 60	Ref		Ref		Ref		Ref	
> 60	1.08 (0.97–1.20)	0.162	1.08 (0.96–1.20)	0.185	1.09 (0.98–1.20)	0.119	1.08 (0.97–1.20)	0.155
**Performance status**								
0	Ref		Ref		Ref		Ref	
1–2	1.48 (1.32–1.65)	< 0.001	1.37 (1.22–1.53)	< 0.001	1.46 (1.32–1.62)	< 0.001	1.35 (1.21–1.50)	< 0.001

To control for confounding, we used a Cox proportional hazards model adjusted for sex, age at diagnosis, performance status, differentiation status, and stage according to TNM classification. We found that only obesity was independently associated with worse OS and DSS compared with those of normal-weight patients. No statistically significant difference was detected between overweight and normal-weight patients. In addition, performance status was also found to be an independent prognostic factor (**Table 2**).

### Prognostic value of BMI in right-sided tumors

We next investigated the prognostic value of BMI among patients with right-sided tumors. Interestingly, the Kaplan–Meier analysis revealed that overweight patients with right-sided tumors had better OS compared with that of normal-weight patients (**Figure 1C**), with an unadjusted HR of 0.80 (95% CI, 0.68–0.93; *p* = 0.004), while the univariate analysis did not find significant differences in OS for obese patients compared with normal-weight or overweight patients (**Figure 1C**). In contrast to the results for left-sided tumors, sex, weight loss, performance status, differentiation status, depth of invasion, node metastasis, and distant metastasis were found to be associated with clinical outcome (**Table 3**, *p* < 0.05). Further evaluation of the effect of BMI on DSS status in right-sided tumors found a similar survival pattern as that in the OS analysis in that overweight patients had better DSS compared with that of normal-weight patients (log-rank test: *p* = 0.001). Beyond the significant association between BMI and DSS, female patients were also found to have lower risk of DSS.

In the multivariate analysis, we utilized the Cox proportional-hazards regression models described in the analysis of left-sided tumors. The results showed that overweight status was significantly associated with prolonged OS and DSS compared with normal-weight status, indicating that overweight status might be a protective factor for patients with right-sided tumors (**Table 3**). However, the difference in OS and DSS between obese and normal-weight patients was not statistically significant (**Table 3**). In contrast to the results obtained for left-sided tumors, female sex and a lesser degree of weight loss were associated with better OS and DSS among right-sided tumors, and these associations reached statistical significance (**Table 3**).

**Table 3:** Associations between BMI and clinical factors with outcome in right-sided tumors.

	Overall survival				Disease-specific survival			
	Unadjusted HR (CI 95%)	*p*	Adjusted HR (CI 95%)	*p*	Unadjusted HR (CI 95%)	*p*	Adjusted HR (CI 95%)	*p*
**BMI**								
Normal weight	Ref		Ref		Ref		Ref	
Overweight	0.80 (0.68–0.93)	0.004	0.82 (0.67–0.92)	0.025	0.68 (0.57–0.78)	< 0.001	0.72 (0.62–0.84)	0.001
Obese	0.92 (0.68–1.04)	0.406	0.95 (0.79–1.15)	0.485	0.91 (0.71–1.15)	0.428	0.89 (0.71–1.11)	0.310
**Weight loss**								
≤ 10%	Ref		Ref		Ref		Ref	
> 10%	1.36 (1.17–1.59)	< 0.001	1.26 (1.03–1.55)	0.025	1.28 (1.06–1.54)	0.010	1.22 (1.04–1.43)	0.013
**Sex**								
Male	Ref		Ref		Ref		Ref	
Female	0.77 (0.67–0.88)	< 0.001	0.78 (0.68–0.90)	0.001	0.83 (0.72–0.96)	0.010	0.82 (0.71–0.95)	0.006
**Age at diagnosis**								
≤ 60	Ref		Ref		Ref		Ref	
> 60	1.07 (0.93–1.22)	0.382	1.10 (0.96–1.27)	0.182	1.07 (0.93–1.23)	0.382	1.10 (0.96–1.27)	0.182
**Performance status**								
0	Ref		Ref		Ref		Ref	
1–2	1.47 (1.29–1.67)	< 0.001	1.31 (1.01–1.70)	0.045	1.42 (1.26–1.61)	< 0.001	1.38 (1.06–1.81)	0.018

### Prognostic value of tumor location stratified by BMI

**Figure 2:**
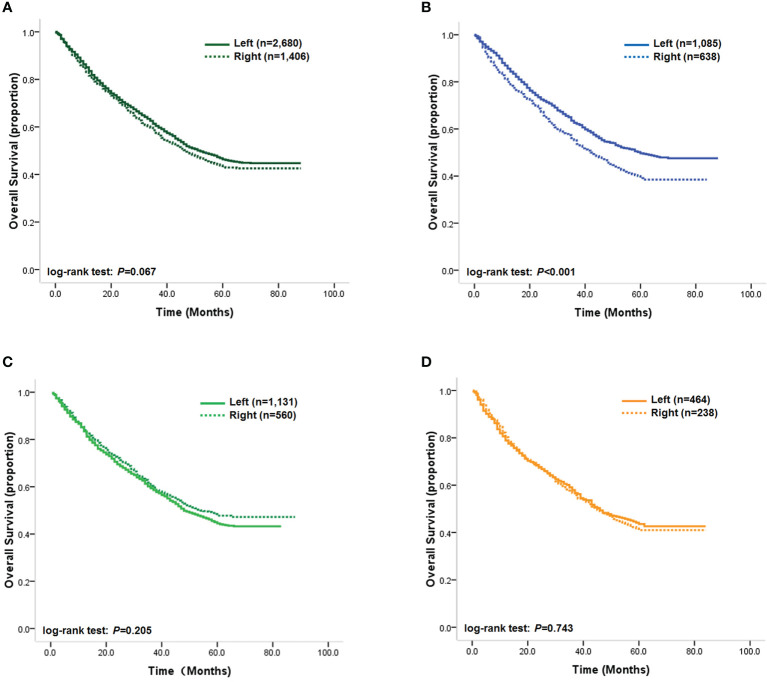
**(A)** Kaplan–Meier survival curves of the overall study cohort; **(B)** Kaplan–Meier survival curves of normal-weight patients; **(C)** Kaplan–Meier survival curves of overweight patients; **(D)** Kaplan–Meier survival curves of obese patients.

Our Kaplan–Meier analysis of the association between tumor location and OS showed that the prognostic difference between right- and left-sided tumors did not reach statistical significance (log-rank test: *p*=0.067, **Figure 2A**). However, as our statistical analysis revealed that the multiplicative interaction of BMI and tumor location was significant for OS, we further analyzed the impact of tumor location on outcomes according to patients’ BMI status in order to reveal how BMI could stratify the prognostic value of tumor location. Our results demonstrated that patients with right-sided tumors achieved statistically significantly unfavorable OS compared with patients with left-sided tumors (log-rank test: *p* < 0.001, **Figure 2B**), with an unadjusted HR of 1.31 (95% CI, 1.15–1.50; *p* < 0.001). Multivariable analysis also showed that primary tumor location was an independent prognostic factor for OS in normal-weight patients, with an adjusted HR for right-sided tumors of 1.28 (95% CI, 1.13–1.46; *p* < 0.001), while differences in DSS remained statistically significant in normal-weight patients, with unadjusted HR and adjusted HRs for right-sided tumors of 1.34 (95% CI, 1.19–1.58; *p* < 0.001) and 1.31 (95% CI, 1.16–1.51; *p* < 0.001), respectively, compared with left-sided tumors. However, a significantly prognostic difference between left- and right-sided tumors was detected in overweight and obese patients (**Figures 2C, D**). These results indicated that tumor location might have opposing effects on patients’ outcomes depending on BMI category.

## Discussion

As an area experiencing a rapid increase in CRC incidence, Asia has seen only a few published studies to date on the prognostic role of BMI on CRC, and these studies’ results have been confusingly discordant with investigations conducted in Western countries (46–49). Recently, tumor location in CRC has been associated with outcomes, although the limited investigations so far have shown conflicting results. This suggests that tumor location might be a powerful confounding factor that could modify both body weight and the risk of death. In addition, the potential impact of BMI on the survival of CRC patients could also be a confounding factor for the prognostic role of tumor location. Therefore, stratifying the analysis according to the tumor location and BMI would be an effective method of addressing the potential bias effect. However, previous work on CRC has omitted the prognostic effect of BMI might be stratified by tumor location, which would lack validity.

We therefore conducted this study to test the hypothesis that there might be interactions between BMI and tumor location in of the context of determining prognosis. We used a study cohort consisting of 4,086 patients from different areas of China. Our results showed that, in addition to an association between tumor location and sex, patients with right-sided tumors were more likely to be of normal weight, which is probably because right-sided tumors cause weight loss. Moreover, tumor location was also found to be associated with tumor differentiation status, in that right-sided tumors were more likely to be poorly differentiated. Although the overall prognosis did not differ according to BMI, our analysis showed a substantial stratifying effect of tumor location on the association of BMI with outcome. Specifically, obese patients with left-sided tumors had significantly unfavorable OS and DSS compared with those of normal-weight patients in both univariate and multivariate analysis, while overweight status was found to only be associated univariately with unfavorable OS in left-sided tumors. Interestingly, among patients with right-sided tumors, overweight patients were found to have more favorable outcomes in both univariate and multivariate analyses compared with normal-weight patients, while the outcome of obese patients with right-sided tumors was not significantly different from that of normal-weight patients. In addition, it was found that the significant association of outcome with sex and weight loss was limited to right-sided tumors. The results of our overall analysis for the total participant cohort are consistent with the CALGB study, which found no association of stage III CRC outcome with BMI status; however, we found statistically significant prognostic differences among BMI groups after we stratified the analysis by tumor location (31). Although the prognostic role of weight change is also different, the weight change in our study is presurgical, while that in the CALGB study took place after adjuvant chemotherapy. Considering the conflicting prognostic role of BMI represented in other studies, which did not stratify by tumor location, our results demonstrate that BMI could adversely impact tumor outcome in CRC patients according to the tumor location.

The specific mechanism by which tumor location stratifies the prognostic value of BMI in CRC might be quite complex. It is most likely related to the host–tumor interaction, which appears to modify tumor cell behavior and corresponding outcome. Increased BMI is associated with a chronic inflammatory state that may elevate circulating factors such as insulin, estrogen, insulin-like growth factor, steroid hormones, and leptin (50–52). These factors may promote a favorable microenvironment for tumor cell survival, proliferation, invasion, and metastasis, thereby heightening risks of tumor recurrence and death. In light of this reasoning, overweight and obesity can be understood to facilitate tumor progression and, hence, poorer outcomes. Although the impact of tumor location on CRC is still controversial, it can cause varying effects on weight loss.

Left- and right-sided tumors differ in their development, clinical presentation, environmental epidemiology, and sex distribution. In addition, recent investigations have found that left-sided tumors are frequently infiltrating, constricting lesions, with a phenotype that involves chromosomal instability, aneuploidy, KRAS, and p53 mutation (53–55). In contrast, right-sided tumors are more likely to be diploid and to be characterized by mucinous histology, high microsatellite instability, CpG island methylation, and BRAF mutations (56, 57). These differences between left- and right-sided tumors could harbor the prognostic value of BMI. In the present study, our results demonstrated that patients with right-sided tumors were more likely to be in a relatively low BMI category, further supporting this notion. In terms of the confounding impact of BMI on the prognostic value of tumor location, we also found that tumor location in patients with different BMI statuses might have opposing prognostic effects.

Our study has several strengths. Our cohort is the largest in Asia to date in the context of investigating prognostic role of BMI, and it is representative of the Chinese patient population. All patients received curative surgical resection and standard adjuvant chemotherapy, and the bias caused by adjuvant chemotherapy was excluded (58). BMI was measured at a uniform time point relative to surgery. In addition, the hospital-based cohort provided detailed data that allowed tumor recurrence and/or the details of the clinical variables to be investigated.

However, the present study had several limitations: we did not consider post-surgical weight loss; we only assessed obesity by BMI without considering other measures of body habitus, such as waist–hip ratio or waist circumference; and in the survival analysis, information related to BMI, such as lifestyle factors, was not accessed or adjusted.

Our findings extend the collective knowledge on the effects of BMI and tumor location beyond their known associations with CRC outcomes by showing that their prognostic role in CRC survivors is stratified by both BMI and tumor location. Although further study is needed to determine the exact mechanism of this stratifying effect, the present study is relevant to efforts to stratify prognostic predictions for CRC patients, with the goals of better management and improved outcomes.

## Data availability statement

The raw data supporting the conclusions of this article will be made available by the authors, without undue reservation.

## Ethics statement

This study was reviewed and approved by the Ethics Committee of Xi’an Jiaotong University. The patients/participants provided their written informed consent to participate in this study.

## Author contributions

DC designed the experimental method and evaluated its feasibility. ZZ, XY, YL, and XG carried out the experiments. WW, BS, YZ, and QH collected the clinical and prognostic information. ZZ wrote the manuscript. DC revised the manuscript. All authors contributed to the article and approved the submitted version.

## Funding

This work was supported by grants from the National Natural Science Foundation of China (grant numbers 81201927, 81672460, 82002957, and 82173337) and the Shaanxi Innovative Talents Promotion Plan (grant numbers 2022TD-58 and 2020JM-391).

## Conflict of interest

The authors declare that the research was conducted in the absence of any commercial or financial relationships that could be construed as a potential conflict of interest.

## Publisher’s note

All claims expressed in this article are solely those of the authors and do not necessarily represent those of their affiliated organizations, or those of the publisher, the editors and the reviewers. Any product that may be evaluated in this article, or claim that may be made by its manufacturer, is not guaranteed or endorsed by the publisher.
